# Physiological and multi-omics responses of *Neoporphyra haitanensis* to dehydration-rehydration cycles

**DOI:** 10.1186/s12870-022-03547-3

**Published:** 2022-04-04

**Authors:** Zekai Wang, Caiping Lu, Juanjuan Chen, Qijun Luo, Rui Yang, Denghui Gu, Tiegan Wang, Peng Zhang, Haimin Chen

**Affiliations:** 1 State Key Laboratory for Managing Biotic and Chemical Threats to the Quality and Safety of Agro-products, Ningbo University, Ningbo, 315211 Zhejiang China; 2 Collaborative Innovation Center for Zhejiang Marine High-efficiency and Healthy Aquaculture, Ningbo University, Ningbo, 315211 Zhejiang China; 3 Zhejiang Mariculture Research Institute, Wenzhou, 325005 China

**Keywords:** *Neoporphyra haitanensis*, Desiccation, Resurrection, Proteome and metabolome

## Abstract

**Background:**

Seaweeds in the upper intertidal zone experience extreme desiccation during low tide, followed by rapid rehydration during high tide. *Porphyra* sensu lato are typical upper intertidal seaweeds. Therefore, it is valuable to investigate the adaptive mechanisms of seaweed in response to dehydration-rehydration stress.

**Results:**

A reduction in photosynthetic capacity and cell shrinkage were observed when *N. haitanensis* was dehydrated, and such changes were ameliorated once rehydrated. And the rate and extent of rehydration were affected by the air flow speed, water content before rehydration, and storage temperature and time. Rapid dehydration at high air-flow speed and storage at − 20 °C with water content of 10% caused less damage to *N. haitanensis* and better-protected cell activity. Moreover, proteomic and metabolomic analyses revealed the abundance members of the differentially expressed proteins (DEPs) and differentially abundant metabolites (DAMs) mainly involved in antioxidant system and osmotic regulation. The ascorbic acid-glutathione coupled with polyamine antioxidant system was enhanced in the dehydration response of *N. haitanensis*. The increased soluble sugar content, the accumulated polyols, but hardly changed (iso)floridoside and insignificant amount of sucrose during dehydration indicated that polyols as energetically cheaper organic osmolytes might help resist desiccation. Interestingly, the recovery of DAMs and DEPs upon rehydration was fast.

**Conclusions:**

Our research results revealed that rapid dehydration and storage at − 20 °C were beneficial for recovery of *N. haitanensis*. And the strategy to resist dehydration was strongly directed toward antioxidant activation and osmotic regulation. This work provided valuable insights into physiological changes and adaptative mechanism in desiccation, which can be applied for seaweed farming.

**Supplementary Information:**

The online version contains supplementary material available at 10.1186/s12870-022-03547-3.

## Background

Water is essential to life [1]. A significant challenge that organisms face when leaving the water and conquering the land is exposure to a dry atmosphere. As one of the most important evolutionary events, desiccation enables terrestrial life to survive outside water. Particularly, some plants can survive under extremely dry conditions, under which they lose more than 90% of cellular water, and resume metabolic activity upon rehydration [2]. Such plants are known as resurrection plants [3]. Today’s terrestrial plants have developed a wealth of structural and functional adaptations for tolerating dry environments. However, these processes are more nuanced for organisms residing in the intertidal environment between the ocean and land. Algae growing in this environment are subject to dry periods dictated by tidal rhythms. Their adaptations to dehydration may be akin to a transitional state of plant evolution. Such desiccation-tolerant algae are found among the red algae (Rhodophyta), brown algae (Phaeophyceae), Chlorophyta, Prasionophyta, and Charophyta families. Algae situated on exposed rocks can tolerate water potentials as low as − 140 MPa for certain periods of time during “complete” desiccation, which is considered to be less than 5% cellular water content [2, 4].

When rehydrated, resurrection plants and intertidal algae can regain their regular metabolic activity. However, the dehydration-rehydration process in intertidal seaweed is very different from that of terrestrial plants. The intertidal zone experiences the rising and falling tides twice daily, leading to its rapid and periodic dehydration-rehydration. In contrast, drought-tolerant terrestrial plants usually recover after long-term dehydration, such as days, months, or even years [2]. Therefore, there are differences in the adaptation models. Some researches on the desiccation tolerance of eukaryotic algae in different tidal zones have revealed that eukaryotic algae can outperform cyanobacteria under mild and non-extreme climatic conditions, and dehydration can reach 96%, with immediate resurrection [2, 4]. Moreover, previous studies have identified several key aspects of the drought tolerance of algae, such as changes in their photosynthetic system. Many algal species from the middle and upper littoral zone increase their photosynthetic rates after some degree of dehydration, and it is believed that light energy is used to fix the bulk of carbons in a short period of time. This process occurs in certain species, such as *Porphyra perforate*, *Fucus distichus*, *Porphyra linearis*, and *Endocladia muricata*. However, the photosynthetic systems of many algal species are quickly shut down when they are dehydrated, and these species include *Bostrychia calliptera*, *Caloglossa leprieurii*, and *Neopyropia haitanensis* [4–7]. Secondly, it is considered that the participation of antioxidant systems will result in the depletion of reactive oxygen species (ROS), and the levels of reduced glutathione (GSH), ascorbic acid (AsA) and vitamin E are increased during dehydration, contributing to the scavenging of free radicals [2]. Wang et al. have reported that Cu/Zn SOD and other antioxidant enzyme genes up-regulated in the genomes of intertidal red algae [8]. In addition, some compatible solutes, such as floridosides, malate acid, succinic acid, and glutamic acid, and several genes encoding serine/threonine kinases and phospholipases may involve in the osmotic tolerance in red algae [9].

*Porphyra* is the seaweed species inhabiting the highest tide zones on global rocky shores. Compared with sublittoral algae, it has stronger drought resistance and rehydration repair efficiency [10, 11]. Furthermore, many types of *Porphyra*, such as *Neopyropia yezoensis* and *N. haitanensis*, are economically important in Asia. The tidally dictated dehydration of these seaweeds is not only a natural phenomenon but also a necessary process for the health of the algae in aquaculture. The benefits of dehydration are reflected in the fact that ultraviolet rays can reduce contaminants on thalli during dehydration, thereby reducing diseases [12, 13]. *Porphyra* can be temporarily stored in cold storage with certain dryness before being returned to the sea to continue to grow [14]. However, there is limited information regarding overall physiological changes (such as water content and photosynthetic capacity), influencing factors, and molecular mechanisms determining the dehydration-rehydration cycle of seaweed.

Herein, we investigated *N. haitanensis*, an economically important seaweed in China. By simulating the dehydration-rehydration cycle, we explored for the effects of different air flow speeds, original water contents, and storage temperatures and time on the recovery and cell viability of *N. haitanensis*, which would be helpful in optimizing the cold storage technology during cultivation. In addition, through proteome and metabolome, we described the differential profiles of *N. haitanensis* proteins and metabolites during the dehydration-rehydration cycle to expound the mechanism of *N. haitanensis* resistance to dehydration-rehydration stress.

## Results

### Effects of different desiccation treatments on dehydration and rehydration of *N. haitanensis*

**Fig. 1:**
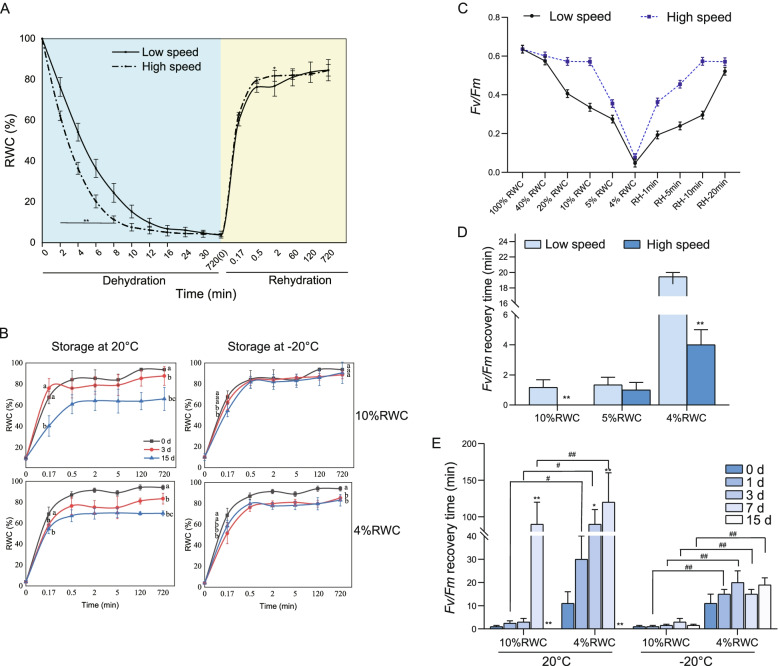
Changes of relative water content and chlorophyll fluorescence curve of *Neoporphyra haitanensis* under dehydration stress. **A** Time course of relative water content (RWC) changes in thalli in the dehydration-rehydration cycle. The thalli were suspended on strings for dehydration stress and followed rehydration process, then harvested at different times to measure RWC. **B** The storage conditions on RWC recovery of thalli after rehydration. The thalli dehydrated with the RWC of 10 and 4% were stored at 20 °C and − 20 °C for 0, 3 and 15 days, and then rehydrated to detect the RWC curve. Different lowercases indicate significant differences, *p* < 0.05, *n* = 10. **C** The photochemical efficiency (*Fv/Fm*) curve of thalli stressed by dehydration-rehydration cycle under air-flow speeds. **D** The *Fv/Fm* recovery time of different dehydrated thalli at low or high air-flow speed. ^**^p < 0.01, comparison between different air flow speed groups. *n* = 10. **E** The *Fv/Fm* recovery time of dehydrated thalli stored at different temperatures. The thalli were suspended on strings for dehydration and rehydration process with low (1.5 m/s) and high (2.5 m/s) air-flow speeds. The thalli with different RWC were stored at 20 or − 20 °C for days, and then the thalli were collected for measuring the *Fv/Fm*. HD (hydration), RH (rehydration). ^*^p < 0.05, ^**^p < 0.01, comparison between different storage time groups; ^#^*p* < 0.05, ^##^*p* < 0.01, comparison between groups with different degrees of drying. *n* = 10

In the present study, we examined the effects of air flow speeds on *N. haitanensis* and found that the rate of water loss was faster for thalli at high air flow speed. The relative water content (RWC) was decreased from 100 to 5% at the high air-flow speed within 12.5 ± 0.1 min, while such duration was 26.3 ± 0.3 min at the low air-flow speed. Once the RWC was decreased to 5%, the thalli were hard to further lose moisture. Even at high air-flow speed, it took 714 min for the RWC to drop from 5 to 4% (Fig. 1A). After rehydration for 2.0 ± 1.0 min, the RWC of the high-speed group was increased and reached 81.79 ± 2.69%, while the RWC of the low-speed group reached 76.28 ± 2.49% (*p* < 0.05). However, the RWC of both groups returned to an almost identical degree after 1 h.

The thalli were subjected to two air-flow speeds to examine the effect of initial RWC on the rehydration ability (Table 1). We found that when the initial RWC was high (> 10% RWC), the air flow speed had little impact on the rehydration rate of thalli, the recovery time was short, and the final RWC was high. Conversely, when the initial RWC was low (≤10% RWC), the time required for the high-speed group to reach the maximum RWC was generally shorter compared with the low-speed group (*p* < 0.05), while the highest RWC at the two speeds was similar (*p* > 0.05).

**Table 1:** The rehydration of *Neoporphyra haitanensis* with different relative water contents at two air-flow speeds

Initial RWC	Initial rehydration rate		The highest RWC (%)		Time to reach the highest RWC (min)	
	Low	High	Low	High	Low	High
40%RWC	2.75^a^	2.77^a^	91.49^a^	92.93^a^	0.5	0.5
20%RWC	3.51^a^	3.43^a^	89.85^a^	92.30^a^	5.0	0.5
10%RWC	3.29^a^	3.60^b^	87.13^a^	89.75^a^	60	10
5%RWC	3.57^b^	3.89^b^	86.74^a^	88.81^a^	60	30
4%RWC	3.30^a^	3.48^a^	84.54^a^	84.53^b^	120	60

*Note*: Different lowercases indicate significant differences, *p* < 0.05, *n* = 10

The thalli were then dehydrated to the RWC of 10 and 4% at low air-flow speed and stored at 20 °C or − 20 °C for different durations. Figure 1B showed that as the storage time was increased, the rehydration rate was gradually decreased. The overall RWC recovery was more significant in the group refrigerated at − 20 °C compared with the 20 °C storage group, although they were lower than fresh samples. The thalli were dehydrated to the RWC of 10% and placed directly into seawater without storage, and an RWC of 90% was achieved. The rehydration rate of this group was significantly greater compared with the 20 °C storage group, which reached 65.96 ± 10.97% after 15 days of storage (*p* < 0.01). When stored at − 20 °C, thalli with an initial RWC of 10% could recover to the RWC of 90.61 ± 10.12% after 15 days of storage, which was not significantly different from the 0-day group (*p* > 0.05). Thalli with an initial RWC of 4% stored at − 20 °C for 15 days still could reach the final RWC of 83.17 ± 5.51% after rehydration. Results indicated that storage at room temperature gradually reduced the rehydration capability of *N. haitanensis*, while refrigeration maintained this ability, and storing the thalli with an initial RWC of 10% led to more significant overall recovery.

### Effects of different dehydration treatments on the photosynthetic activity of *N. haitanensis*

Dehydration caused a rapid drop in *Fv/Fm* regardless of the air flow speed, while it dropped faster in the high-speed group. When the RWC of the high- and low-speed groups was decreased from 100 to 5%, the time for *Fv/Fm* to drop to the lowest point (0.04) was 12.5 ± 0.1 and 26.3 min, respectively. However, thalli in the high-speed group could return to a score of 0.57 within 10 min of rehydration, while the low-speed group only returned to 0.29 (Fig. 1C). With the decrease of the initial RWC, the two air-flow speeds required different durations to return to a normal *Fv/Fm* value. The recovery time of the high-speed group was significantly shorter (*p* < 0.01). However, it took 18.5 min to recover the *Fv/Fm* to a normal level in the low-speed group with an initial RWC of 4%, which was significantly higher than the 5% RWC group (Fig. 1D), because it required more time to decrease the RWC from 5 to 4%.

*N. haitanensis* dehydrated to the RWC of 10% or 4% was stored at 20 °C and − 20 °C. During the 15 days of storage, the *Fv/Fm* recovery time of thalli stored at 20 °C was gradually increased with the extension of the storge, while this value in the − 20 °C group was not significantly affected by the storage time (*p* > 0.05, Fig. 1E). The recovery of the *Fv/Fm* of thalli stored at the RWC of 10% was better than that of the 4% RWC group (*p* < 0.05). At 20 °C, the *Fv/Fm* recovery time of thalli with an RWC of 10% stored for 7 days was 90 times greater than that of the samples without storage (0 day) (*p* < 0.01), and the *Fv/Fm* could not be fully recovered after 15 days of storage. When thalli with an RWC of 4% were stored at 20 °C for 7 days, it took 120 min (*p* < 0.01) for the *Fv/Fm* to reach 0.5, and it did not return to its original value after 15 days. However, the *Fv/Fm* of the cold storage groups was resumed to normal values ​​in quickly. Although the 4% RWC group was more difficult to recover than the 10% RWC group, it could be resumed within 20 min.

### Effects of dehydration and rehydration on cellular states of *N. haitanensis*

**Fig. 2:**
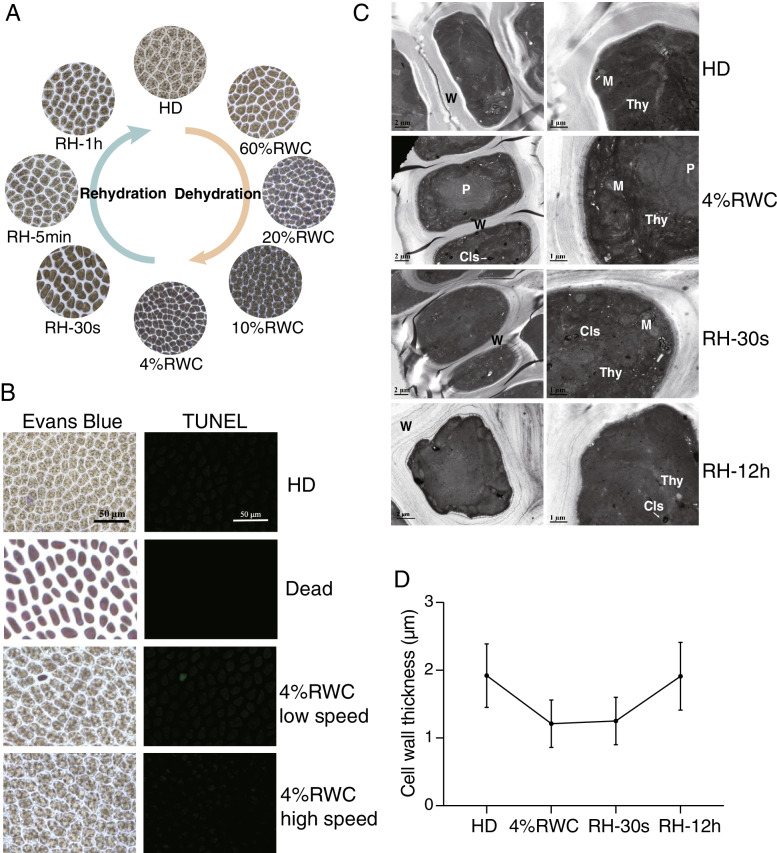
*Neoporphyra haitanensis* morphology after exposure to dehydration-rehydration cycle*.* **A** Overview of thalli cells for the dehydration-rehydration cycle (× 400). **B** Cell viability of dehydrated thalli at different air-flow speeds. Samples were dehydrated to the RWC of 4% at low or high air-flow speeds, and then harvested for staining with Evans Blue or subjected to TUNEL assay for the detection of apoptotic-like cell death (× 400). **C** Transmission electron micrographs of thalli cells of HD (hydration), 4% RWC and RH (rehydration) for 30 s and 12 h. Scale bars: 2 μm and 0.5 μm. **D** The cell wall thickness of different dehydrated and rehydrated cells

By observing the cellular changes of *N. haitanensis* during the dehydration and rehydration processes, we found that with the loss of water, the cell gap was gradually decreased, the cell size was also affected, showing noticeable shrinkage, and the cell color became slightly darker (Fig. 2A). When the dehydrated thalli were placed into the seawater, the cell gap and size were gradually recovered. After 1 h of rehydration, the cell size was fully recovered. The Evans blue staining method was used to observe cell death during dehydration, while the TUNEL staining was used to observe the degree of cell apoptosis. Figure 2B showed that most of the cells in thalli dehydrated to the RWC of 4% at low air-flow speed survived, and very few apoptosis occurred, while no dead or apoptotic cells were found at high air flow speed. Transmission electron microscopy (TEM) showed that the cell wall (W) of the HD group was relatively loose, the double membrane structure was clear, the internal organelle structure was apparent, the thylakoids (Thy) were arranged uniformly, the mitochondria (M) were short rod-shaped and the internal cristae were clear. However, at the RWC of 4%, *N. haitanensis* shrank, exhibiting compact and wrinkled cell walls. The plasma membrane was close to the cell wall with no plasmolysis (Fig. 2C, D). The number of cristae in the mitochondria was reduced, the arrangement was disordered, the shape was irregular, and the arrangement of chloroplast thylakoids was loose but not degraded. Moreover, there were multiple vesicle-like concentric layered structures (Cls). After 30 s of rehydration, the overall cell morphology was expanded, the internal structure of mitochondria began to recover, and there were still vesicle-like Cls. After 12 h of rehydration, the cell membrane was not recovered to normal level, and a few vesicles were still in the Cls. The internal organelles of the cell were intact and clear, the organelle membranes were clear, the thylakoids were evenly distributed, the mitochondrial double membrane structure was complete, the cell wall returned to a loose state and the cell wall thickness returned to the value of the HD group (Fig. 2C, D).

The storage experiments using dehydrated thalli showed that at 20 °C, thalli with the RWC of 10% were alive after 24 h, while some cells were positive for TUNEL staining after 3 days but negative for Evans blue staining. This finding indicated the cells were undergoing apoptosis, while no cells died. After 7 days, cell death and apoptosis were increased. When stored for 15 days, more than half of the cells were positive for Evans blue staining and there were some cells in an apoptotic state. Therefore, it was proved that storage at − 20 °C had protective effects on thalli, and there was no evidence of cell death/apoptosis even after 15 days of storage (Fig. S1A). The pattern of cell death in thalli with an RWC of 4% was similar to that of the 10% RWC group, except that after 15 days of storage at 20 °C, almost all the cells died, while under cold storage conditions cell death and apoptosis were controlled (Fig. S1B).

### Proteomics and metabolomics analyses of the dehydration-rehydration cycle in *N. haitanensis*

**Fig. 3:**
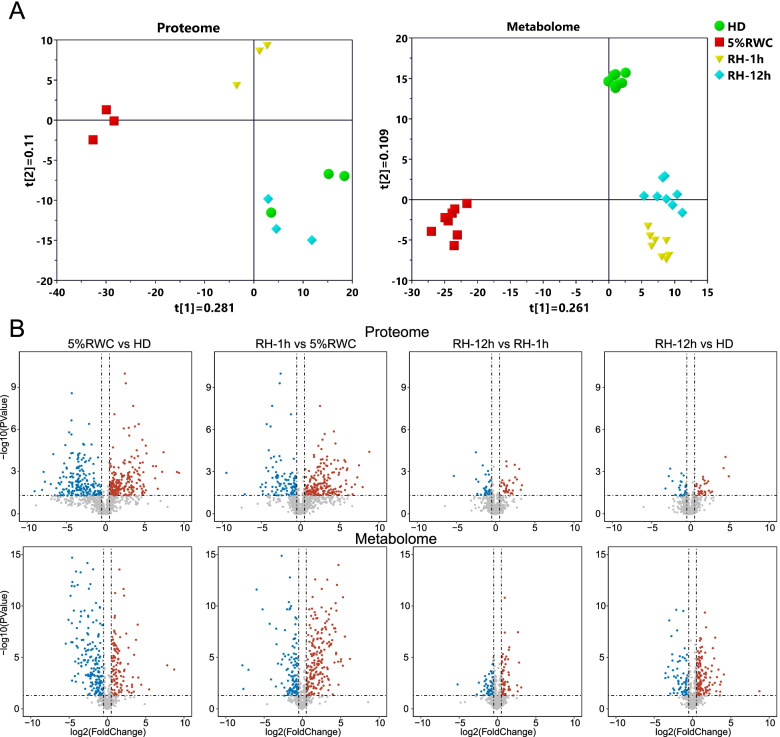
Distribution and trend of metabolites and proteins in the dehydration-rehydration cycle of *Neoporphyra haitanensis*. **A** Partial least squares discriminant analysis (PLS-DA) showed the distribution of four groups of 1117 proteins and 597 metabolites. The same color points indicate biological replicates for each treatment. **B** Volcano plots of differentially expressed proteins (DEPs) and differentially abundant metabolites (DAMs). The threshold of significantly down-regulated (blue dots, fold change ≤0.667) and up-regulated proteins and metabolites (red dots, fold change ≥1.5) are highlighted

To investigate the adaptation to intertidal environments under the dehydration-rehydration cycle of *N. haitanensis*, we performed a comparative proteomics analysis using the Orbitrap LC-MS to obtain the expressed proteins among dehydrated algae with the RWC of 5% and rehydrated algae for 1 h and 12 h (RH-1 h and RH-12 h). Based on the MS analysis of the quantification results, 1117 proteins were identified with the unique peptide ≥1. The expression levels of proteins analyzed by partial least squares discriminant analysis (PLS-DA) showed the high variability in protein contributions to dehydration and rehydration responses (Fig. 3A). The HD, 5%RWC, and RH-1 h groups were separated, especially the 5% RWC group, while the cluster of the RH-12 h group was very close to the HD group. Furthermore, as the rehydrated time was increased up to 12 h, the cluster of RH-12 h was nearly close to the cluster HD, reflecting that the protein changes of the RH-12 h group were almost recovered to those of HD, which was consistent with the periodical physiological changes of *N. haitanensis*.

Based on the high-performance liquid chromatography coupled with mass spectrometry (HPLC-MS) and gas chromatography-mass spectrometry (GC-MS) analyses, we identified 597 metabolites in HD, 5%RWC, RH-1 h, and RH-12 h groups. The normalized peaks and the pareto-scaled data of metabolites were also analyzed by PLS-DA (Fig. 3A). The HD, dehydration and rehydration groups were separated entirely, indicating that the metabolites of dehydration and rehydration were significantly different from the HD group. Especially in the rehydration period, it showed a trend of time-dependent cluster formations that gradually approached the HD group. The results reflected that the metabolites changed in the dehydration-rehydration cycle had similar trends to those of proteins, although it was still different from the HD group after 12 h of rehydration.

**Fig. 4:**
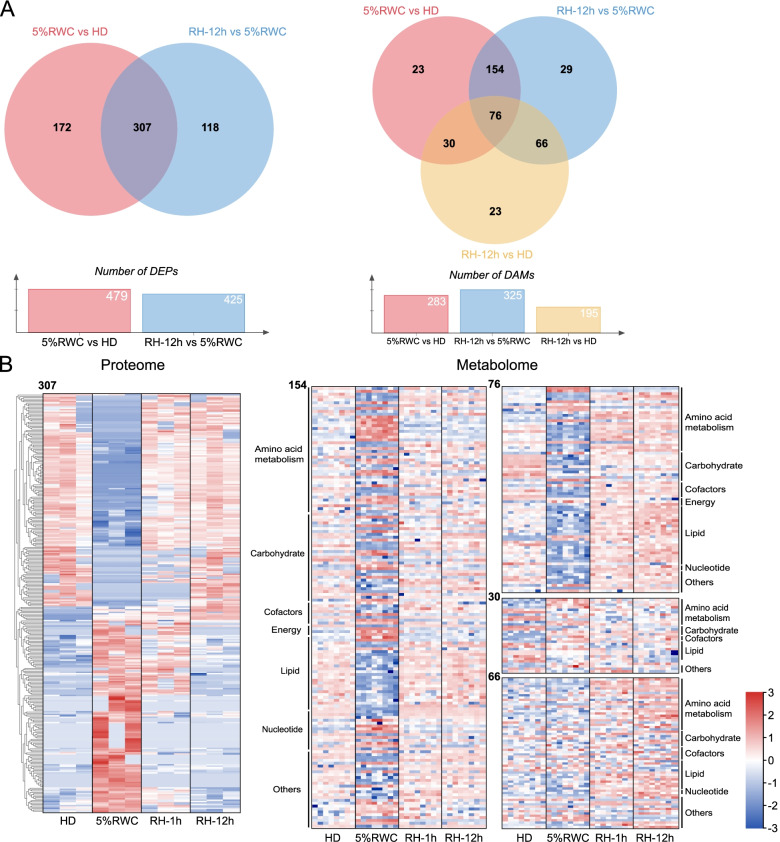
Dynamic response of differentially expressed proteins (DEPs) and differentially abundant metabolites (DAMs) in dehydration-rehydration cycle of *Neoporphyra haitanensis*. **A** Venn diagram showed the overlaps of DEPs and DAMs in the process of dehydration-rehydration circle. **B** Heatmap of proteome showed the hierarchical clustering and abundance of shared DEPs during the dehydration-rehydration cycle. Heatmaps of metabolome displaying the classification and abundance of shared DAMs in the four overlaps. The shared DEPs and DAMs were obtained based on the overlaps from Venn diagrams. Heatmaps were constructed based on the normalized intensities of proteins (*n* = 3) and metabolites (*n* = 8)

Fold change [FC] ≥ 1.5 or ≤ 0.667, and *p*-value < 0.05 were used to screen out differentially expressed proteins (DEPs) and differentially abundant metabolites (DAMs). We characterized the significantly up- and down-regulated proteins and metabolites by volcano plots during each dehydration/rehydration process (Fig. 3B). Obviously, there were many DEPs and DAMs in “5%RWC vs HD” group and “RH-1h vs 5%RWC” group, and the extent of the difference was significantly reduced in the rehydration from 1 h to 12 h. Besides, “RH-12h vs HD” group also showed certain DAMs. Venn diagrams were used to reveal the overlaps of DEPs and DAMs in the dehydration-rehydration circle (Fig. 4A). The overlap of DEPs between “5%RWC vs. HD” and “RH-12h vs. 5%RWC” groups was 64%, with 307 shared DEPs. The hierarchical clustering and visualization of these shared DEPs on the heatmaps showed that the expressions of half of them were up-regulated during dehydration and recovered to the levels of the HD group after 12 h of rehydration, while the others showed an opposite trend (Fig. 4B). The classification and abundance of 154, 76, 30, and 66 shared DAMs in the four overlaps among “5%RWC vs. HD”, “RH-12 h vs. 5%RWC”, and “RH-12h vs. HD” were shown in heatmaps (Fig. 4B). The 230 shared DAMs between “5%RWC vs. HD” and “RH-12 h vs. 5%RWC” groups dramatically responded to the dehydration process, and 154 DAMs were completely recovered after rehydration for 12 h. In comparison, 76 DAMs still had difference by comparing with HD levels.

### KEGG pathway analysis of DEPs and DAMs in the dehydration-rehydration cycle

**Fig. 5:**
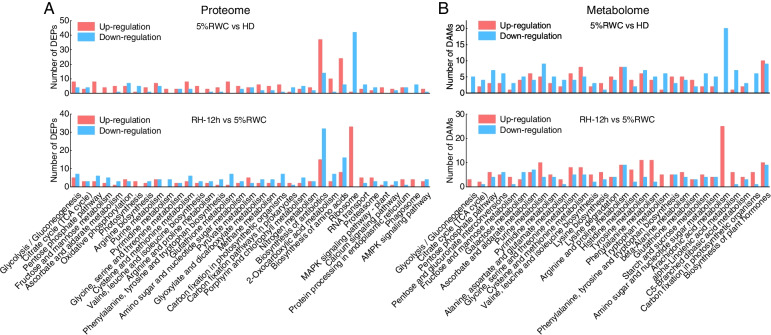
KEGG pathway analysis of differentially expressed proteins (DEPs) and differentially abundant metabolites (DAMs) in dehydration-rehydration cycle of *Neoporphyra haitanensis*. **A** The numbers of DEPs in KEGG pathways. **B** The numbers of DAMs in KEGG pathways. **“**5%RWC vs. HD” represented dehydration, and “RH-12h vs. 5%RWC” represented rehydration**.** The x-axis represented different KEGG pathways, which were annotated by KEGG [15]. The y-axis represented the number of DEPs/DAMs

We further analyzed the function of DEPs and DAMs using the KEGG pathway annotation method [15]. A total of 35 pathways mapping in KEGG of DEPs were obtained, which were mainly involved in “photosynthesis”, “carbon fixation in photosynthetic organisms”, “glutathione metabolism”, and “ascorbate and aldarate metabolism” (Fig. 5A). Moreover, 30 pathways mapping in KEGG of DAMs were obtained, especially “carbon fixation in photosynthetic organisms”, “arachidonic acid metabolism”, “glutathione metabolism” and “ascorbate and aldarate metabolism” (Fig. 5B). Table S1 lists the normalized intensities and FCs of typical DEPs and DAMs.

**Fig. 6:**
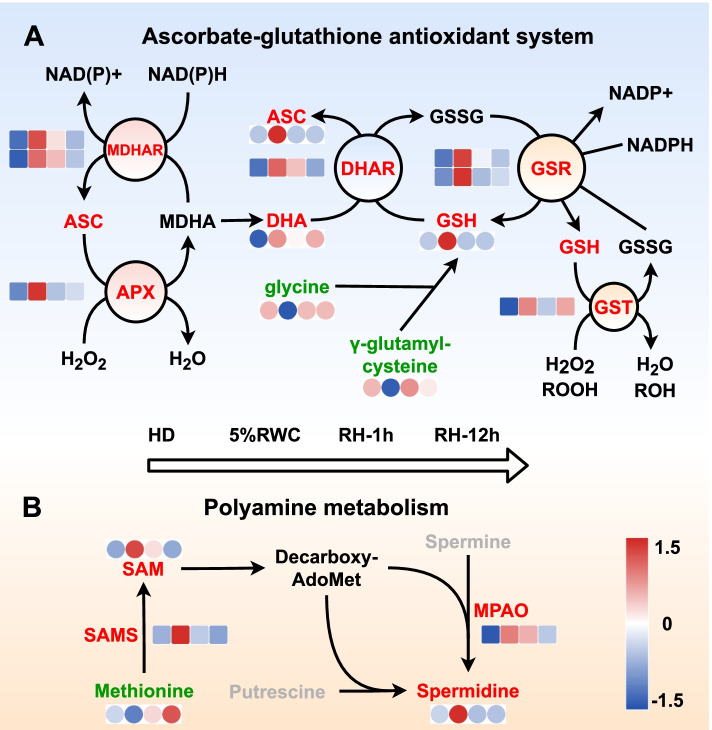
Overview of important pathways in the dehydration-rehydration cycle of *Neoporphyra haitanensis*. The pathways were reconstructed based on KEGG pathways. **A** Ascorbate-glutathione antioxidant pathway. **B** Polyamine metabolism. The square and circular heatmaps showed the normalized intensities of proteins (*n* = 3) and metabolites (*n* = 8), respectively. The heatmap data of each protein or metabolite showed the regulation from the HD (hydration), 5% RWC, RH-1 h and RH-12 h groups (from left to right)

It was noticed that the AsA-GSH antioxidant system was significantly affected (Fig. 6A). “Glutathione metabolism”, and “ascorbate and aldarate metabolism” pathways involved in antioxidant processes had more up-regulated DEPs and DAMs in dehydration, which were down-regulated in rehydration. The levels of ascorbate peroxidase (APX), glutathione dehydrogenase (DHAR), glutathione reductase (GSR), glutathione S-transferase (GST) and monodehydroascorbate reductase (MDHAR) were enhanced about 1.51-11.00 folds, and the levels of dehydroascorbic acid (DHA), AsA and GSH were increased by 1.61, 433.59 and 45.13 folds in dehydration, respectively. Besides, the contents of the precursors of GSH, glycine, and γ-glutamylcysteine had opposite changing trends compared with GSH. Moreover, we also observed dramatic changes correlated with polyamine metabolic pathway. Three polyamines were observed in the metabolome. The level of spermidine (SPD) was up-regulated by 5.66 folds in dehydration, while spermine (SPM) and putrescine (PUT) were remained unchanged. The levels of precursor S-adenosylmethionine (SAM) were increased about 6.07 folds. Still methionine was decreased 37.62% in the 5% RWC group, accompanied by the up-regulated levels of polyamine oxidase (MPAO) and S-adenosylmethionine synthetase (metk) proteins in this pathway about 2.16 and 1.63 folds, respectively (Fig. 6B). Interestingly, DAMs in the “arachidonic acid metabolism” pathway obviously showed a reducing trend to decrease in the dehydration process. The levels of C20 and C18 belonging to the oxygenated derivatives of unsaturated fatty acids oxylipins were all decreased in the 5%RWC group and then recovered to the levels of the HD group in rehydration (Fig. S2).

### The carbohydrate changes of *N. haitanensis* in the dehydration-rehydration cycle

**Fig. 7:**
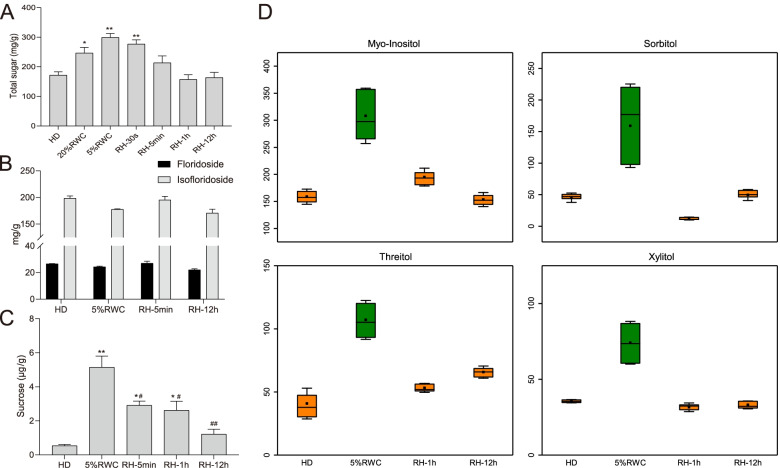
Response of osmoregulation substances in the dehydration-rehydration cycle of *Neoporphyra haitanensis*. **A** Total contents of soluble sugars. **B** The contents of floridoside and isofloridoside. **C** The contents of sucrose. All the data were shown as the means ± SD for three biological replicates. HD (hydration), RH (rehydration). ^*^p < 0.05, ^**^p < 0.01, compared to HD group; ^#^*p* < 0.05, ^##^*p* < 0.01, compared to 5% RWC group. **D** Box plots of normalized intensities of four polyols in dehydration-rehydration cycle. The *y*-axis in box indicated the minimum, 25 percentiles, mean (closed squares), median (−–), 75 percentile and maximum of all the replication from bottom to top

The total soluble sugar levels in dehydrated thalli were increased to 1.74 times that of fresh thalli, once the RWC levels were reduced to 5% (Fig. 7A, *p* < 0.01). The increment of (iso)floridosides in response to dehydration was not observed (Fig. 7B, *p* > 0.05), which is the most abundant low-molecular-weight carbohydrate (LMWC) in Bangiophyceae and plays a significant role in osmotic pressure regulation [16]. In addition, trehalose was not detected in thalli, while sucrose concentration was increased by 9.57 folds (Fig. 7C, *p* < 0.01) following dehydration. Nevertheless, such concentrations of sucrose (μg/g) were not sufficient for mitigating desiccation tolerance. Intriguingly, the levels of four polyols, including myo-inositol, sorbitol, threitol, and ribose, were increased by 1.94-2.96 folds in the 5%RWC group compared with the HD group (Fig. 7D), which had dramatically higher normalized intensity compared with sucrose (Table S1). After rehydration, the above polyol metabolism-related proteins and metabolites were down-regulated to nearly recovering to the levels of HD group.

## Discussion

*Porphyra* sensu lato, as seaweeds inhabiting in the high tide zone on seaside rocks, will be exposed to the air and dehydrated at low tide. The dehydration rate is directly affected by air flow speed. Because of the monolayer of the thallus, the rate of water loss is very fast, which can even be further accelerated at high air-flow speed. It can lose more than 90% of its water content in 10 min, indicating that *N. haitanensis* does not have a particular water retention barrier to slow down the rate of water loss, such as a gelatinous layer or mucus.

When *N. haitanensis* was dehydrated, thallus cells shrank rapidly, intercellular gaps were reduced, cell walls contracted, and the chloroplast thylakoid structure was loosened. However, the cell wall and membrane remained intact, and all the changes could be restored after rehydration. When the green algae *Ulva compressa* lose water, the outer layer of the cell wall remains intact, while the inner layer is wavy [17], which is the same as the changes in the cell wall of *N. haitanensis*. It is believed that the pectin matrix in the cell wall of resurrection plants can maintain the cell wall plasticity during dehydration [18]. The cell wall of *Porphyra* is composed of porphyrans and cellulose. Changes in the flexible and rigid cell wall structure help prevent mechanical damage caused by cytoplasm shrinkage. Although *N. haitanensis* thylakoids are loosely arranged after dehydration, the membrane structure remains intact, indicating that although dehydration will cause certain damages to the thylakoids, it will not cause chloroplast disintegration and such damages can be quickly recovered after rehydration.

Generally, when plants are dehydrated due to stress, the photosynthetic rate will decrease [2]. However, some algae, such as *P. perforate*, *N. yezoensis*, and *Pelvetia fastigiata f. gracilis* can increase the photosynthetic capability when dehydrated to a certain degree [5, 6]. Furthermore, *P. linearis* exposed to the air will increase its photosynthesis to the maximum rate when its RWC drops to 60% [4]. However, the mechanism by which slight dehydration increases the photosynthetic rate is currently unclear. It is generally believed that the loss of the water film on the surface of the algae reduces the CO_2_ diffusion barrier [19]. However, in the present study, the *Fv/Fm* was rapidly decreased to the bottom in the 5%RWC group, suggesting that the photosynthetic system was shut-down. The same phenomenon has also been observed in other intertidal seaweeds, such as *Bostrychia calliptera* and *Caloglossa leprieurii* [7]. The experiments presented herein revealed that the decrease in *Fv/Fm* was directly related to the dehydration speed (air-flow speed), indicating that *Fv/Fm* was closely related to the total content of water.

When the tide returns, *Porphyra* needs to be returned to the water quickly to restore photosynthetic activity to a normal state, which is a conventional ability of intertidal seaweeds. In general, the photosynthetic activity of seaweeds recovers quickly within 2 h of submersion [19], while the specific time required and the degree of recovery vary with the vertical distribution of seaweeds. Ji et al. have found that algae living in high tide zones such as *N. yezoensis* and *Ishige okamurae* can recover 100% of their photosynthetic ability after 1 h of rehydration [20]. However, species living in the low tide zone or sub-tidal zones such as *Ulva pertusa*, *Chondrus ocellatus*, *Ahnfeltia paradoxa* and *Gelidium elegans* cannot recover completely [20]. Here, we observed that the water content and *Fv/Fm* recovered very quickly after rehydration, reaching the RWC of 81.79 ± 2.69% within 2 min and normal *Fv/Fm* ​​values within 20 min, indicating that the rehydration and recovery abilities of *N. haitanensis* were very powerful. In addition, we observed that the degree of dehydration, air-flow speed, and duration had a great impact on the recovery of RWC and the photosynthetic capacity of intertidal seaweeds. It has been reported that when cells of *P. linearis* lose water excessively or dry out too long, the rate and extent of photosynthetic recovery will decrease [4]. *Gastroclonium coulteri* dehydrated to 35% every day can fully restore their photosynthetic activity. While continuous dehydration to 50% daily or 70% once results in unrestorable photosynthetic capacity [21]. We also identified that the time required for the 4% RWC thalli to restore photosynthetic efficiency was much longer compared with the 10% RWC group. The speed of dehydration has a great influence on the speed of recovery. High air flow speed produces rapid dehydration, while the degree and speed of rehydration also accelerate, and the recovery speed of *Fv/Fm* is much greater compared with those dehydrated at a low air-flow speed. Another significant finding was that the duration and storage conditions after dehydration also had a great influence on recovery after rehydration. In Asia, *N. haitanensis* and *N. yezoensis* are both economically cultivated seaweeds. During the breeding process, there is cold storage of seeding net technology, meaning that the seedlings that grow on the nets can be temporarily stored under a frozen condition for a period of time (months, even years) after they are dehydrated to a certain degree of moisture, before being returned to the sea for further cultivation [14]. Therefore, this experiment explored the rehydration recovery of *N. haitanensis* with different water contents after being stored at two temperatures for different durations. It was identified that recovery after storage at room temperature was significantly affected by storage duration. Not only the recovery time for photosynthetic capacity was significantly increased, but also the viability could not be restored after 15 days of storage and RWC only returned to 65.96 ± 10.97%. However, storing the seaweed at − 20 °C not only fully recovered the RWC, but also the cells did not die and the *Fv/Fm* was not greatly affected. Even after storage for 15 days, cells still could return to their initial viability within 20 min. Secondly, storage at specific water contents also had a great impact on the recovery efficiency. For example, when stored with the RWC of 10% at room temperature for 3 days, seaweed restored to the normal state within 3 min. At − 20 °C, even if stored for 15 days, the seaweed restored within 1.5 min, and there was no cell death. In farming, gametophytes on the nets will be dehydrated to the RWC of 10-20% and then stored under frozen conditions. Our results here explained the rationality of this technique. In addition, we observed that the dehydrated *N. haitanensis* survived well when stored in a refrigerator for more than a year after rehydration.

One of the harmful effects of dehydration is damage to the photosynthetic system. Similar results have been obtained from a previous publication [22–25], suggesting the high inhibition caused by dehydration on the photosynthetic light reaction and carbon fixation of *N. haitanensis*, which can be attributed to the desiccation resistance (Table S1). Besides, after dehydration, chlorophyll molecules continue to be excited, while energy not used in carbon fixation will cause the formation of singlet oxygen, inevitably leading to oxidative damage caused by photo-inhibition [26]. Therefore, the activation of the antioxidant system is a necessary means of protection for plants or algae that are exposed to drought [2]. Thiol-redox regulation of GSH pathway is the main antioxidant pathway of plants, such as *Ramonda serbica*, during dehydration and rehydration [27–29]. The contents of peroxides and the activities of glutathione oxidases of *Pleopeltis polypodioides* are increased in dehydration, and then lowered after rehydration [30]. As general non-enzymatic antioxidants, the metabolites of the AsA-GSH cycle can effectively eliminate ROS under stressful conditions [31, 32]. The synthesis of AsA and GSH may be a major means for *N. haitanensis* to attenuate oxidant damages. The down-regulated glycine and γ-glutamylcysteine, GSH’s precursors, were supposed to synthesize GSH. Then, enzymes, such as MDHAR, DHAR, and GSR, and metabolite DHA were continuously up-regulated during the dehydration process, which was beneficial to the rapid regeneration of the final products AsA and GSH. The redox reaction of AsA and GSH via APX and GST can help *N. haitanensis* eliminate ROS caused by dehydration and reduce damages to ensure that algae can be restored to the normal growth state after rehydration.

Polyamines are the only organic polycations existing in sufficient quantities in cells [33] and can interact with negatively charged molecules, such as DNA, protein or membrane phospholipids to protect essential proteins and DNA and stabilize the plasma membrane [34, 35]. Polyamines also have antioxidative activity when combined with metal ions, such as Fe^2+^ and Cu^2+^, to prevent them from providing electron donors for ROS formation [34]. Remarkably, changes in PA titers are broadly correlated with drought resistance traits, and high levels of polyamines via the overexpression of SAM synthetase will enhance the plant tolerance [36]. In intertidal red algae *Gracilaria corticata*, the decrease in ROS production is concurrent with the significantly higher levels of free PAs under desiccation [37]. The protective molecular SPD of *Haberlea rhodopensis* and *Ramalina farinacea* has higher contents in desiccation condition than in rehydration condition [38, 39]. It is consistent with our results that SPD, one of polyamine, and its synthase polyamine oxidase MPAO were both increased under dehydration, which might play a role in antioxidation for the repair of the desiccation-induced damage. Overall, after rehydration, the down-regulated important antioxidants, AsA, GSH, and SPD, were gradually recovered to the levels of the HD group, indicating that *N. haitanensis* could rapidly restore as the environment returned to normal.

Organic osmolytes, such as non-reducing sugar, polyols, free amino acids and their derivatives are found in all water-stressed systems in plants to reduce the osmotic potential in the cells to maintain turgor pressure [40]. In the dehydration process, osmolytes can alleviate water loss, reduce cell damage, and provide sufficient time for the biosynthesis of desiccation tolerance substances [41]. Non-reducing sugars, such as sucrose and trehalose, have been repeatedly mentioned in the desiccation tolerance, where sucrose is the primary carbohydrate in tissues of resurrection plants and green algae upon desiccation [32, 42]. The levels of total soluble sugar in thallus cells were increased by 1.74 folds compared with fresh thalli once the RWC levels were reduced to 5%. Intriguingly, (iso)floridoside represents the primary sink for photosynthetic carbon in red algae and serves as an osmolyte that should accumulate to high levels under stressful conditions [43]. However, its level was not increased in *N. haitanensis* during dehydration. Cao et al. have also observed that the content of (iso)floridoside in *Porphyra umbilicalis* is not changed in response to desiccation [44]. Therefore, it is reasonable that (iso)floridoside, the primary product of carbon assimilation, should not increase when the photosystem is inhibited. However, in our previous study, we have reported that (iso)floridoside levels in *N. haitanensis* are significantly increased after 2 h of desiccation [45]. In the present study, it took less than 30 min for RWC to decrease to 5% in thalli, suggesting that (iso)floridoside was not an osmotic pressure regulator that could quickly respond to environmental changes. In addition, trehalose was not detected in *N. haitanensis* cells. Although the μg/g concentration of sucrose was increased by 9.57 times following dehydration, such concentrations were not sufficient for mitigating desiccation tolerance. However, several polyols, such as myo-inositol, sorbitol, threitol, and ribose, were dramatically increased in dehydration, playing a role in osmotic regulation. A similar phenomenon has been observed in terrestrial green algae *Trebouxiophyceae*, showing that the accumulations of glycerol, erythritol, ribitol, arabitol, mannitol, and sorbitol under osmotic and desiccation stresses form an integral part of a biochemical protective strategy against water loss [46, 47]. And the increased polyols of *Selaginella lepidophylla* also can decelerate the rate of water loss in dehydration and slow down the adsorption in rehydration [48]. Besides, myo-Inositol, sorbitol, threitol and ribose are C4-C6 molecules, demanding lower energy for production compared with disaccharides (C12). The biosynthesis of sucrose requires 109 ATP equivalents, while sorbitol only requires 57 ATP equivalents [47]. Due to the multiple functions of these polyols as organic osmolytes, compatible solutes, antioxidants, heat protectants, and respiratory substrates, and finite energy as the photosynthesis was shut down under dehydration, it seemed that *N. haitanensis* tended to accumulate energetically cheaper organic osmolytes for resisting desiccation. This might be the reason why low-molecular-weight polyols accumulated under dehydration replaced disaccharides as organic osmolytes in *N. haitanensis*. Moreover, polyols could readily be restored to the levels of the HD group within 1 h of rehydration, indicating that the speed of osmotic regulation of *N. haitanensis* was sensitive and rapid.

## Conclusions

*N. haitanensis*, a high-tide seaweed, can withstand water loss up to 96% and survive rehydration. When extreme desiccation occurred, their photosynthetic system was shut down (as exhibited by a fall in *Fv/Fm*), cells shrank, and organelle structures were loosened. After rehydration, RWC and photosynthetic capacity could be restored, while this was affected by the degree of dehydration, as well as the speed and duration of dehydration. Rapid dehydration and storage at − 20 °C could protect the cellular state of *N. haitanensis*, which was conducive to rehydration and survival. Multi-omics analysis suggests that the GSH, AsA, and SPD as typical antioxidants were activated during dehydration, playing an essential protective role in reducing the damage of desiccation to cells. Besides, polyols, as energetically cheaper organic osmolytes, might replace disaccharides for osmotic regulation under dehydration. The recovery time course of these metabolites and proteins revealed that *N. haitanensis* could adapt to extreme desiccation. Collectively, our study provided new insights into the periodic desiccation adaptation of *N. haitanensis* in the intertidal zone. Moreover, we also offered some technical parameters for cold storage net technology in economically important seaweed farming.

## Methods

### Materials

*N. haitanensis* cultivar ZD-1 (developed by our group and registered by the Ministry of Agriculture and Rural Affairs, P. R. China) was collected from the Experimental Station at the coast of Xiangshan Harbor in Zhejiang Province, China (121°57′5″E, 29°46′30″N). Prof Qijun Luo performed the formal identification of the plant material. Healthy thalli with a length of 15-20 cm were selected, washed with sterilized seawater, and exposed to 0.7% KI solution for 5-10 min. After rinsing three times at 22 ± 2 °C, samples were exposed to 40 μmol·m^− 2^ s^− 1^ of light under a photoperiod of 12 h: 12 h (L:D) before the follow-up experiments.

### Dehydration and rehydration treatments

*N. haitanensis* thalli of similar length and width were divided into 10 groups (*n* = 10). After the fresh weight was measured, they were hung on strings to simulate dehydration stress with at an air temperature of 22 ± 2 °C, relative humidity of 70 ± 5% and light irradiation of 40 μmol·m^− 2^ s^− 1^ with a photoperiod of 12 h: 12 h (L:D) at low air-flow speed of 1.5 m/s or high speed of 2.5 m/s. The dehydrated thalli were immersed in sterile seawater for rehydration. The samples were weighed every 2 min during this process. Thalli with different water contents or collected at various rehydration time points were used to detect rehydration rates, photosynthetic parameter changes, and cell morphology and viability. Samples were divided into the following groups: hydrated samples (HD), samples with the RWC of 60, 20, 10, 5, and 4%, samples rehydrated for 30 s (RH-30s), 5 min (RH-5 min), 1 h (RH-1 h), and 12 h (RH-12 h). The samples were snap-frozen in liquid nitrogen and then stored at − 80 °C. The HD group, 5%RWC group, RH-1 h group, and RH-12 h group were selected for omics analysis. Eight biological replicates were used for the LCMS-based and GCMS-based metabolomic analyses. Three biological replicates were used for the proteomic analysis.

The relative moisture content of thalli was determined using the formula as follows: RWC (%) = (F_w_ − D_w_)/(F_Tw_ − D_w_) × 100, where F_w_ is the weight of thalli after dehydration for a specific time, D_w_ represents the dry weight after incubation at 80 °C for 6 h, and F_Tw_ is the fresh thallus weight [3].

Thalli of the 10% RWC and 4% RWC groups were placed into polyethylene bags, which were then sealed and stored at 20 °C and − 20 °C for 1, 3, 7, and 15 days to determine of rehydration rates, fluorescence parameter (*Fv /Fm*), and cell status.

### Photosynthetic parameter measurement

After 10 min of dark adaptation, the chlorophyll fluorescence parameter of *N. haitanensis* was measured using WATER-PAM (Walz, Germany). The minimum fluorescence *F*_*0*_ was obtained by measuring light at an intensity of 0.1 μmol·m^− 2^·s^− 1^ and a pulse frequency of 1 Hz applied for 0.8 s. Next, the maximum fluorescence Fm was measured with a saturation pulse of 4000 μmol·m^− 2^·s^− 1^. Subsequently, the chlorophyll fluorescence parameters *Fv/Fm* =  (*Fm*-*F*_*0*_)/*Fm* were obtained.

### Cell death and apoptosis measurement

Briefly, the thalli on the slide were stained with 0.5% Evans blue for 10 min in the dark, and then rinsed with sterile seawater. The slides were observed under a fluorescence microscope (ECLIPSE Ti-U, Nikon, Japan). Dead cells were stained blue.

According to the manufacturer’s instructions, apoptosis was assessed using a one-step TUNEL apoptosis assay kit from Beyotime Biotechnology Company (Shanghai, China). After treatment, the slides were sealed with an antifade mounting medium and observed under a fluorescence microscope (ECLIPSE Ti-U, Nikon, Japan).

### Ultrastructural studies

The samples were pre-fixed in a pre-cooled 4% glutaraldehyde fixative solution for 1 h, followed by sequential fixation in 2.5% glutaraldehyde at 4 °C overnight and 2.5% glutaraldehyde+ 2% paraformaldehyde. Subsequently, samples were immersed in 0.1 M PBS (PH 7.0) three times 15 min each. Next, samples were incubated in 1% osmium acid at 4 °C in a dark place for 2 h before a second PBS wash. Thalli were then dehydrated using a gradient of ethanol concentrations, followed by exposure to anhydrous acetone twice for 20 min. The specimens were then incubated in absolute acetone and embedded in epoxy resin, followed by sectioning with a LEICA EM UC7 ultratome (Leica, Germany). Finally, sections were stained with 1% uranyl acetate and 1% lead citrate and examined using TEM (H-7650, Hitachi, Japan).

### Analysis of total soluble sugar and LMWC

Fresh samples (50 mg) were ground in liquid nitrogen, followed by extraction using 1 mL of 80% hot ethanol three times (15 min each). The mixtures were centrifuged at 3000 r/min to obtain the supernatant. A 40 μL supernatant was mixed with 20 μL of 5% phenol solution and 100 μL sulfuric acid, and the absorbance of the mixture was measured at a wavelength of 492 nm. Total sugar contents of the samples were calculated based on standard curves. Six biological replicates were conducted per treatment.

Another 100 mg of each sample was extracted with 1 mL of 70% ethanol using a homogenizer. The extraction solution was centrifuged at 5000 rpm for 30 s, and this process was repeated three times, followed by centrifugation at 5000 rpm for 15 s for six cycles. Before analysis, supernatants were then collected, dried, and dissolved in 50 mL acetonitrile/H_2_O. (Iso)floridosides, sucrose, and trehalose were quantified using a Finnigan Surveyor and a TSQ Quantum Access system (Thermo Fisher Scientific, Pittsburgh, PA, USA). (Iso)floridoside analyses were conducted as previously described by Chen et al. [49]. Specifically, calibration curves for (iso)floridoside quantification were constructed using the standard compounds directly extracted from *N. haitanensis*. Analyses of sucrose and trehalose were performed at 35 °C using an Acquity BEH Amide (2.1 mm × 150 mm, 1.7 μm) column. The flow rate was set at 0.15 mL/min, and the injection volume was 3.0 μL. The elution gradient consisted of ultrapure water (solvent A) and acetonitrile (0.1% solvent B), with both containing 0.1% ammonium hydroxide. Mobile phase B was changed from 20 to 45% after 12 min, then decreased to 20%, and held for 3 min. The ionization conditions were set as sheath gas pressure (N_2_) flow rate of 25 L/min, auxiliary gas pressure (N_2_) flow rate of 5 Abs, spray voltage of 2.5 kV, vaporizer temperature of 300 °C, and capillary temperature of 350 °C. The collision gas pressure was set at 1.5 mTorr. HPLC-MS was operated in the negative electrospray ionization (ESI) mode with selected reaction monitoring for quantification. The calibration curves for sucrose and trehalose quantification were then plotted with the standards (Sigma-Aldrich, St. Louis, MO, USA).

### Metabolomic analysis based on HPLC-MS and GC-MS

The samples (200 mg) were placed into a 2-mL EP tube containing 0.6 mL cold methanol and 2-chlorophenylalanine (4 μg/mL), and then the mixture was vortexed for 30 s. After that, 100 mg glass beads were added into the sample tube and transferred into the TissueLysis II tissue grinding machine (Scientz, China) for grinding 60 s at 25 Hz. To collect the supernatant, the homogenized mixture was ultrasonicated for 15 min at room temperature and centrifuged for 10 min at 1750 g. Then, each supernatant was filtrated through a 0.22-μm membrane for HPLC-MS analysis. Finally, 20 μL of the supernatant from each sample was mixed as the quality control (QC) samples.

The sample extracts were analyzed using the HPLC-MS system (HPLC, Thermo UltiMate 3000; MS, Thermo Q Exactive). HPLC separation was equipped with an ACQUITY UPLC® HSS T3 (150 × 2.1 mm, 1.8 μm, Waters) column, and the column temperature was maintained at 40 °C. The mobile phase consisted of 5 mM ammonium formate in water (A) and acetonitrile (B) for negative mode, or 0.1% formic acid in water (C) and 0.1% formic acid in acetonitrile (D) for positive mode. The flow rate was 0.25 mL/min. The injection volume was 2 μL. An increasing linear gradient of solvent B/D (v/v) was used as follows: 0 ~ 1 min, 2% B/D; 1 ~ 9 min, 2% ~ 50% B/D; 9 ~ 12 min, 50% ~ 98% B/D; 12 ~ 13.5 min, 98% B/D; 13.5 ~ 14 min, 98% ~ 2% B/D; 14 ~ 17 min, 2% B-negative model (14 ~ 20 min, 2% D-positive model). The ESI was carried out at the spray voltage of 3.8 kV and − 2.5 kV in positive and negative modes, respectively. Sheath gas and auxiliary gas were set at 30 and 10 arbitrary units, respectively. The capillary temperature was 325 °C. The analyzer scanned over a mass range of mass-to-charge ratio (m/z) 81-1000 for a full scan at a mass resolution of 70,000. Data-dependent acquisition (DDA) MS/MS experiments were performed with a higher-energy C-trap dissociation (HCD) scan. The normalized collision energy was 30 eV. Dynamic exclusion was implemented to remove unnecessary information in MS/MS spectra.

The samples (50 mg) were placed into a 2-mL EP tube, followed by the addition of 1 mL cold methanol: chloroform: water (5:2:2, v/v/v). Next, 100 mg glass beads and 100 μL ribosanol (20 μg/mL) were added into the tube, followed by homogenization for 2 min at 30 Hz. The mixture was allowed to stand at 4 °C for 3 min and then vortexed for 30 s. The above-mentioned procedure was repeated twice. The homogenized mixture was centrifuged at 12000 g for 10 min at 4 °C. Subsequently, 50 μL supernatant from each sample was mixed as the QC samples. The remaining supernatant dried with a vacuum concentrator. For derivatization, 80 μL of 20 mg/mL methoxyamine pyridine solution was added to dissolve the dried samples, and the mixture was vortexed for 30 s and reacted at 80 °C for 30 min. Finally, 100 μL Bis(trimethylsilyl)trifluoroacetamide (BSTFA) reagent containing 1% chlorotrimethylsilane (TMCS) was added into the samples, and the reaction was maintained at 70 °C for 90 min, followed by centrifugation at 12,000 rpm for 3 min. The supernatant was transferred to the injection bottle for GC-MS analysis.

GC-MS was performed on the GC coupled with MS system (GC, Agilent 7890B; MS, LECO Pegasus BT) using a DB-5MS capillary column (30 m × 250 μm i.d., 0.25 μm film thickness, Agilent J & W Scientific, Folsom, CA, USA) at a constant flow of 1 mL/min helium. Briefly, 1 μL of the sample was injected in a split mode at a split ratio of 10:1 by the auto-sampler. The injection temperature was 280 °C. The temperature of the transfer line ion source was 320 °C and 230 °C. The temperature program was set as follows. The initial temperature was held at 50 °C for 0.5 min, and then it was increased up to 320 °C at an increment of 15 °C/min and maintained at 320 °C for 9 min. The MS was determined by the full-scan method within a range of m/z 75- 650.

### Proteomics analysis based on HPLC-MS

Each freeze-dried sample (200 mg) was ground into fine powder with pestle and mortar in liquid nitrogen. Proteins were prepared by SDT lysis buffer [2% sodium dodecyl sulfate (SDS), 100 mM dithiothreitol (DTT), and 100 mM tris(hydroxymethyl)-aminomethane hydrochloride (tris-HCl), pH 7.6] and quantified by the BCA protein quantitative Kit (Fisher Scientific, USA). Subsequently, the proteins were precipitated by acetone and digested with trypsin (Promega, USA). Finally, the peptide was passed through a desalting column (sep-Pak C18, 1 cc, 100 mg, Waters, USA) to remove the salt and then freeze-dried.

The freeze-dried peptides were reconstituted with 50 μL 0.1% formic acid and analyzed using an Orbitrap Fusion Lumos MS (Thermo Scientific, USA) coupled online to a nanoscaled liquid chromatography system (Easy-nLC 1200, Thermo Fisher Scientific, USA). Chromatographic separation was equipped with a series of columns, including a peptide capture column (Acclaim PepMap C18, 100 μm × 20 mm, Thermo Scientific) and a peptide analysis column (Acclaim PepMap C18, 75 μm × 250 mm, Thermo Scientific). The HPLC mobile phase was 0.1% formic acid (A) and acetonitrile/water solution (8:2, v/v) containing 0.1% formic acid (B). An increasing linear gradient was used as follows: 0 ~ 3 min, 10% ~ 13% B; 3 ~ 42 min, 13% ~ 29% B; 42 ~ 53 min, 29% ~ 37% B; 53 ~ 54 min, 37% ~ 95% B; 54 ~ 60 min, 95% B. The column flow rate was 600 nL/min, with a typical injection volume of 5 μL.

The mass spectrometer ion source spray voltage was 2.2 kV, the temperature of the ion transfer tube was 320 °C, and the data-dependent mode was adopted to automatically switch between MS and tandem MS (MS/MS) to collect data. The full scan was carried out using Orbitrap mass with a resolution set to 120,000 (m/z, 200), and the scanning range was m/z 350-1550. The maximum ion introduction time was 50 ms, the automatic gain control (AGC) was set to 4e^5^ ions, and then 32% of the HCD was used to fragment the precursor ions meeting the MS/MS fragmentation conditions and scan with Orbitrap, and the scanning resolution was set to 15,000. The scanning range was automatically controlled according to the m/z ratio of the precursor ion, and the minimum scanning range was fixed at m/z = 100. The minimum ionic strength for MS/MS was set to 50,000. The maximum ion introduction time for MS/MS was 22 ms, the AGC control was set to 4e^5^ ions, and the precursor ion selection window was set to 1.6 Da. MS/MS collection was carried out for ions with 2-7 charge numbers, the dynamic exclusion was set to be that after one MS/MS collection for each parent ion, and no MS/MS collection was carried out for the same parent ion within 30 s.

### Data processing

The metabolomics data were converted into mzXML format through Proteowizard software (https://proteowizard.sourceforge.io/) for HPLC-MS and MSD software (Agilent, USA) for GC-MS. Peak identification, filtration and alignment were achieved by the XCMS package of R language (www.r-project.org) to obtain the information of m/z, retention time (RT), and intensity. Then, the accurate molecular weight and MS/MS fragments were used to identify metabolite structures by matching them with annotations in online and company-owned databases. The intensities were proceeded by internal standard normalization for GC-MS and batch normalization for HPLC-MS for quantitation. The normalized intensities were autoscaled by mean-centering and scaled to unit variance. LabelFree quantitative proteomics technique was utilized to search the original data by Sequest embedded in Proteome Discoverer 2.2 (Thermo Scientific). The protein prediction was achieved by the RNA-Seq database to obtain the qualitative and quantitative information of peptides and proteins. The median of normalized intensities for protein quantitation was utilized to eliminate the errors caused by sample volume and instrument operation.

PLS-DA was performed to predict and descript modelling and discriminative variable selection by SIMCA-P software (www.umetrics.com). Discrimination two classes of samples was achieved by a statistically significant threshold of Student’s *t*-test (*p* < 0.05). Box plots and heatmaps were constructed with origin (www.originlab.com) and TBtools (https://github.com/CJ-Chen/TBtools/releases). KEGG pathway was annotated by KAAS software (www.genome.jp/tools/kaas/) and metaboanalyst (www.metaboanalyst.ca).

### Statistical analysis

For each physiological experiment, three biological replicates were prepared for each group. The results were presented as the mean ± standard deviation (SD). In addition, statistical significance was evaluated based on the Student’s *t*-test and Pearson correlation analysis. Data acquired were analyzed using SPSS version 16.0. A *p* < 0.05 was considered statistically significant.

## Supplementary Information

**Additional file 1: Figure S1.** The effect of storage condition on rehydration viability of desiccated thalli. The thalli with the RWC of 10% (A) or 4% (B) were storage under 20 or − 20 °C for different time, and then harvested for Evans Blue staining and TUNEL assay (× 400).

**Additional file 2: Figure S2.** Response of oxylipins in the dehydration-rehydration cycle of *Neoporphyra haitanensis*. Heatmaps showed the abundance of oxylipins in dehydration-rehydration cycle. Heatmaps were constructed based on the normalized intensities of metabolites (*n* = 8).

**Additional file 3: Table S1.** The normalized intensities and fold-changes of typical differentially expressed proteins and differentially abundant metabolites.

## Data Availability

The metabolomic dataset analyzed was available in the MetaboLights databases under MTBLS3968 / https://www.ebi.ac.uk/metabolights/MTBLS3968. The proteomic dataset was available in the ProteomeXchange databases under the PRIDE data set PXD030324 / https://www.ebi.ac.uk/pride/archive/projects/PXD030324.

## References

[1] Scharwies JD, Dinneny JR (2019). Water transport, perception, and response in plants. J Plant Res.

[2] Lüttge U, Beck E, Bartels D. Plant desiccation tolerance. Berlin: Springer-Verlag Berlin Heidelberg; 2011.

[3] Xu Z, Xin T, Bartels D, Li Y, Gu W, Yao H (2018). Genome analysis of the ancient tracheophyte *Selaginella tamariscina* reveals evolutionary features relevant to the acquisition of desiccation tolerance. Mol Plant.

[4] Lipkin Y, Beer S, Eshel A (1993). The ability of *Porphyra linearis* (Rhodophyta) to tolerate prolonged periods of desiccation. Bot Mar.

[5] Oates BR (1986). Components of photosynthesis in the intertidal saccate alga *Halosaccion americanum* (Rhodophyta, Palmeriales). J Phycol.

[6] Gao K, Aruga Y (1987). Preliminary studies on the photosynthesis and respiration of *Porphyra yezoensis* under emersed conditions. J Tokyo Univ Fish.

[7] Peña EJ, Zingmark R, Nietch C (1999). Comparative photosynthesis of two species of intertidal epiphytic macroalgae on mangrove roots during submersion and emersion. J Phycol.

[8] Wang DM, Yu XZ, Xu KP, Bi GQ, Cao M, Zelzion E (2020). *Pyropia yezoensis* genome reveals diverse mechanisms of carbon acquisition in the intertidal environment. Nat Commun.

[9] Sun M, Zhu Z, Chen J, Yang R, Luo Q, Wu W (2019). Putative trehalose biosynthesis proteins function as differential floridoside-6-phosphate synthases to participate in the abiotic stress response in the red alga *Pyropia haitanensis*. BMC Plant Biol.

[10] Blouin NA, Brodie JA, Grossman AC, Xu P, Brawley SH (2011). *Porphyra*: a marine crop shaped by stress. Trends Plant Sci.

[11] Brawley SH, Blouin NA, Ficko-Blean E, Wheeler GL, Lohr M, Goodson HV (2017). Insights into the red algae and eukaryotic evolution from the genome of *Porphyra umbilicalis* (Bangiophyceae, Rhodophyta). P Natl Acad Sci USA.

[12] Wang WJ, Li XL, Sun TQ, Liang ZR, Liu FL, Sun XT (2019). Effects of periodical drying and non-drying on nutrient content and desiccation tolerance of an intertidal *Pyropia yezoensis* strain subject to farming conditions. J Appl Phycol.

[13] Li X-l, Wang W-j, Liu F-l, Liang Z-r, Sun X-t, Yao H-q (2018). Periodical drying or no drying during aquaculture affects the desiccation tolerance of a sublittoral *Pyropia yezoensis* strain. J Appl Phycol.

[14] Watanabe Y, Morikawa T, Mine T, Kawamura Y, Nishihara GN, Terada R (2017). Chronological change and the potential of recovery on the photosynthetic efficiency of *Pyropia yezoensis* f. *narawaensis* (Bangiales) during the sporelings frozen storage treatment in the Japanese Nori cultivation. Phycol Res.

[15] Kanehisa M, Goto S (2000). KEGG: kyoto encyclopedia of genes and genomes. Nucleic Acids Res.

[16] Karsten U, West JA, Zuccarello GC, Engbrodt R, Yokoyama A, Hara Y (2003). Low molecular weight carbohydrates of the Bangiophycidae (Rhodophyta). J Phycol.

[17] Holzinger A, Herburger K, Kaplan F, Lewis LA (2015). Desiccation tolerance in the chlorophyte green alga *Ulva compressa*: does cell wall architecture contribute to ecological success?. Planta.

[18] Ahl LI, Mravec J, Jorgensen B, Rudall PJ, Ronsted N, Grace OM (2019). Dynamics of intracellular mannan and cell wall folding in the drought responses of succulent *Aloe* species. Plant Cell Environ.

[19] Davison IR, Pearson GA (1996). Stress tolerance in intertidal seaweeds. J Phycol.

[20] Ji Y, Gao KS, Tanaka J (2005). Photosynthetic recovery of desiccated intertidal seaweeds after rehydration. Prog Nat Sci.

[21] Hodgson LM (1981). Photosynthesis of the red alga *Gastroclonium conlteri* (Rhodophyta) in response to changes in temperature, light intensity, and desiccation. J Phycol.

[22] Huang LB, Peng LN, Yan XH (2021). Multi-omics responses of red algae *Pyropia haitanensis* to intertidal desiccation during low tides. Algal Res.

[23] Chen H, Chu JS-C, Chen J, Luo Q, Wang H, Lu R (2021). Insights into the ancient adaptation to intertidal environments by red algae based on a genomic and multiomics investigation of *Neoporphyra haitanensis*. Mol Biol Evol.

[24] Barbato R, Tadini L, Cannata R, Peracchio C, Jeran N, Alboresi A (2020). Higher order photoprotection mutants reveal the importance of DeltapH-dependent photosynthesis-control in preventing light induced damage to both photosystem II and photosystem I. Sci Rep.

[25] Townsend AJ, Ware MA, Ruban AV (2018). Dynamic interplay between photodamage and photoprotection in photosystem II. Plant Cell Environ.

[26] Zakar T, Laczko-Dobos H, Toth TN, Gombos Z (2016). Carotenoids assist in cyanobacterial photosystem II assembly and function. Front Plant Sci.

[27] Sgherri C, Stevanovic B, Navari-Izzo F (2004). Role of phenolics in the antioxidative status of the resurrection plant *Ramonda serbica* during dehydration and rehydration. Physiol Plant.

[28] Kranner I, Beckett R, Hochman A, Nash TH (2008). Desiccation-tolerance in lichens: a review. Bryologist.

[29] Oliver MJ, Guo LN, Alexander DC, Ryals JA, Wone BWM, Cushman JC (2011). A sister group contrast using untargeted global metabolomic analysis delineates the biochemical regulation underlying desiccation tolerance in *Sporobolus stapfianus*. Plant Cell.

[30] John SP, Hasenstein KH (2018). Biochemical responses of the desiccation-tolerant resurrection fern *Pleopeltis polypodioides* to dehydration and rehydration. J Plant Physiol.

[31] Blomstedt CK, Griffiths CA, Gaff DF, Hamill JD, Neale AD (2018). Plant desiccation tolerance and its regulation in the foliage of resurrection "Flowering-Plant" species. Agronomy.

[32] Dinakar C, Bartels D (2013). Desiccation tolerance in resurrection plants: new insights from transcriptome, proteome, and metabolome analysis. Front Plant Sci.

[33] Alcazar R, Altabella T, Marco F, Bortolotti C, Reymond M, Koncz C (2010). Polyamines: molecules with regulatory functions in plant abiotic stress tolerance. Planta.

[34] Shi J, Fu XZ, Peng T, Huang XS, Fan QJ, Liu JH (2010). Spermine pretreatment confers dehydration tolerance of citrus in vitro plants via modulation of antioxidative capacity and stomatal response. Tree Physiol.

[35] Schweikert K, Burritt DJ (2015). Polyamines in Macroalgae: Advances and Future Perspectives. J Phycol.

[36] He MW, Wang Y, Wu JQ, Shu S, Sun J, Guo SR (2019). Isolation and characterization of S-Adenosylmethionine synthase gene from cucumber and responsive to abiotic stress. Plant Physiol Biochem.

[37] Kumar M, Gupta V, Trivedi N, Kumari P, Bijo AJ, Reddy CRK (2011). Desiccation induced oxidative stress and its biochemical responses in intertidal red alga *Gracilaria corticata* (Gracilariales, Rhodophyta). Environ Exp Bot.

[38] Unal D, Senkardesler A, Sukatar A (2008). Abscisic acid and polyamine contents in the lichens *Pseudevernia furfuracea* and *Ramalina farinacea*. Russ J Plant Physl+.

[39] Gechev TS, Benina M, Obata T, Tohge T, Sujeeth N, Minkov I (2013). Molecular mechanisms of desiccation tolerance in the resurrection glacial relic *Haberlea rhodopensis*. Cell Mol Life Sci.

[40] Yancey PH (2005). Organic osmolytes as compatible, metabolic and counteracting cytoprotectants in high osmolarity and other stresses. J Exp Biol.

[41] Kalapos T (2003). Desiccation and survival in plants. Drying without dying. Community Ecol.

[42] Holzinger A, Karsten U (2013). Desiccation stress and tolerance in green algae: consequences for ultrastructure, physiological and molecular mechanisms. Front Plant Sci.

[43] Barbier G, Oesterhelt C, Larson MD, Halgren RG, Wilkerson C, Garavito RM (2005). Comparative genomics of two closely related unicellular thermo-acidophilic red algae, *Galdieria sulphuraria* and *Cyanidioschyzon merolae*, reveals the molecular basis of the metabolic flexibility of *Galdieria sulphuraria* and significant differences in carbohydrate metabolism of both algae. Plant Physiol.

[44] Cao Y, Ashline DJ, Ficko-Blean E, Klein AS (2020). Trehalose and (iso)floridoside production under desiccation stress in red alga *Porphyra umbilicalis* and the genes involved in their synthesis. J Phycol.

[45] Qian FJ, Luo QJ, Yang R, Zhu ZJ, Chen HM, Yan XJ (2015). The littoral red alga *Pyropia haitanensis* uses rapid accumulation of floridoside as the desiccation acclimation strategy. J Appl Phycol.

[46] Gustavs L, Gors M, Karsten U (2011). Polyol Patterns in Biofilm-Forming Aeroterrestrial Green Algae (*Trebouxiophyceae*, *Chlorophyta*). J Phycol.

[47] Gustavs L, Eggert A, Michalik D, Karsten U (2010). Physiological and biochemical responses of green microalgae from different habitats to osmotic and matric stress. Protoplasma.

[48] Yobi A, Wone BWM, Xu WX, Alexander DC, Guo LN, Ryals JA (2013). Metabolomic profiling in *Selaginella lepidophylla* at various hydration states provides new insights into the mechanistic basis of desiccation tolerance. Mol Plant.

[49] Chen JJ, Song D, Luo QJ, Mou T, Yang R, Chen HM (2014). Determination of floridoside and isofloridoside in red algae by high-performance liquid chromatography–tandem mass spectrometry. Anal Lett.

