# Supplementary figures and images for: Physiological and multi-omics responses of Neoporphyra haitanensis to dehydration-rehydration cycles
Source: BMC Plant Biol. 2022 Apr 4;22:168. doi: 10.1186/s12870-022-03547-3 (PMC8978406; doi:10.1186/s12870-022-03547-3)

**A**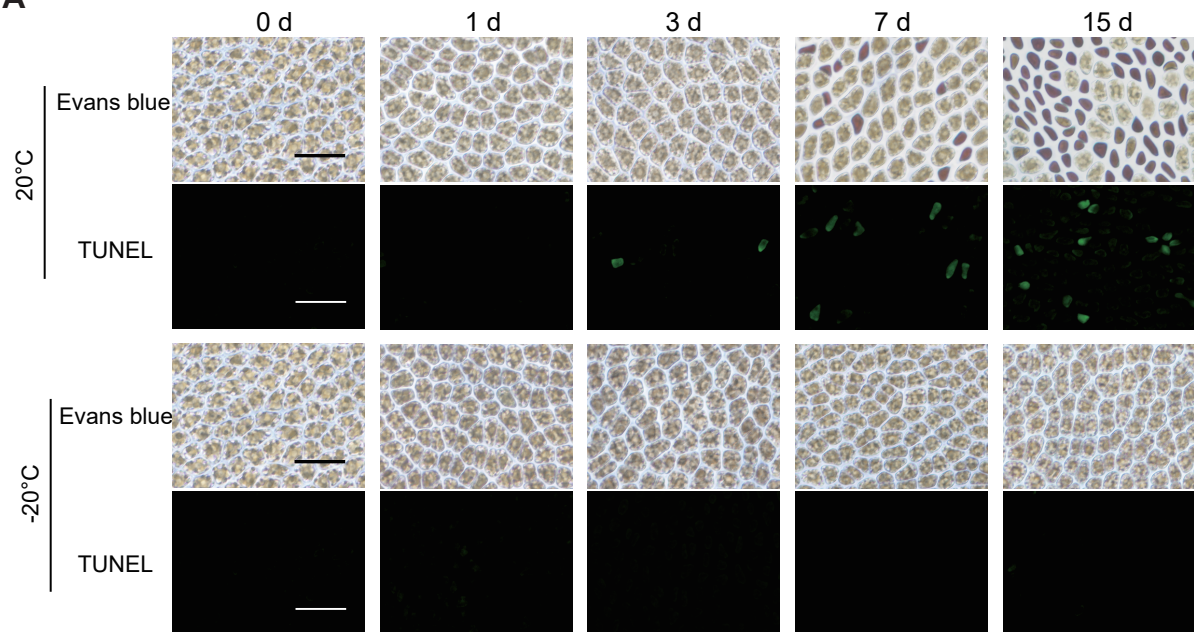**B**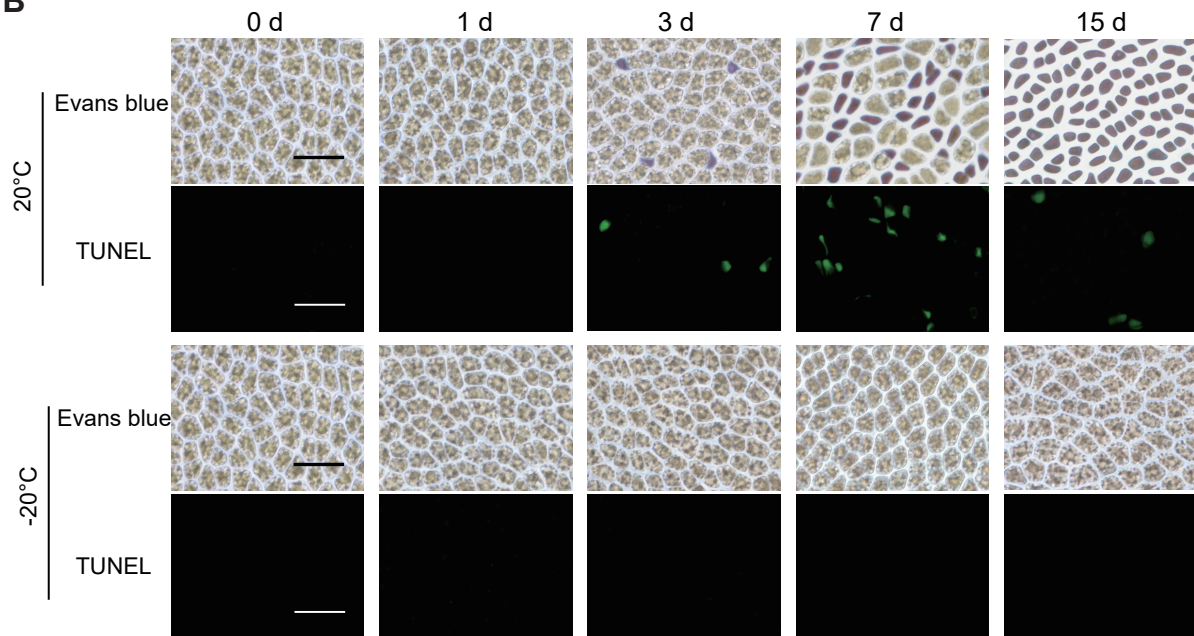

Supplement: Supplementary file 1 — Additional file 1: Figure S1. The effect of storage condition on rehydration viability of desiccated thalli. The thalli with the RWC of 10% (A) or 4% (B) were storage under 20 or − 20 °C for different time, and then harvested for Evans Blue staining and TUNEL assay (× 400). [file 12870_2022_3547_MOESM1_ESM.pdf]

## Eicosanoids (C20)

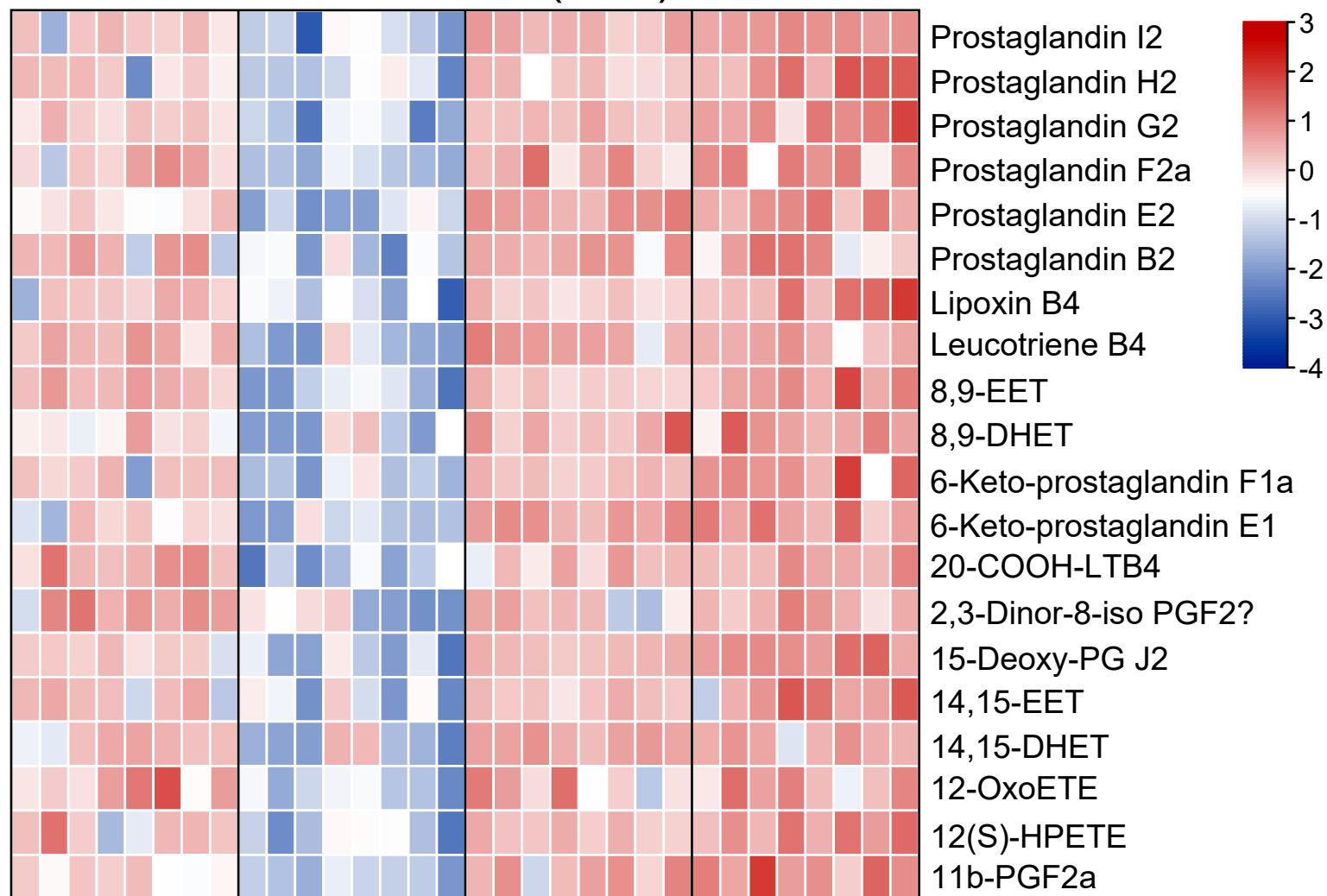

## Octadecanoids (C18)

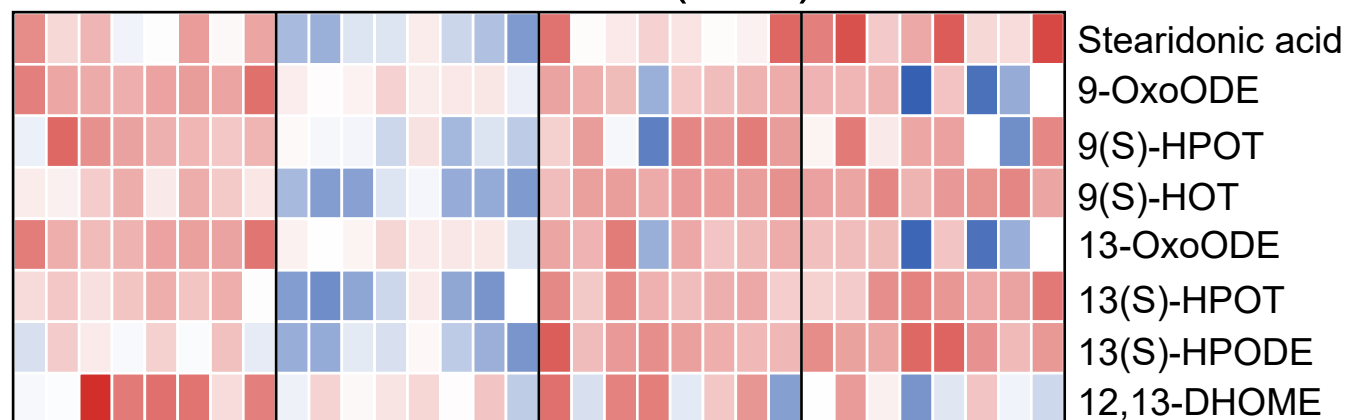

HD

5%RWC

RH-1h

RH-12h

Supplement: Supplementary file 2 — Additional file 2: Figure S2. Response of oxylipins in the dehydration-rehydration cycle of Neoporphyra haitanensis. Heatmaps showed the abundance of oxylipins in dehydration-rehydration cycle. Heatmaps were constructed based on the normalized intensities of metabolites (n = 8). [file 12870_2022_3547_MOESM2_ESM.pdf]
