# Supplementary material for: Physiological and multi-omics responses of Neoporphyra haitanensis to dehydration-rehydration cycles
Source: BMC Plant Biol. 2022 Apr 4;22:168. doi: 10.1186/s12870-022-03547-3 (PMC8978406; doi:10.1186/s12870-022-03547-3)
Supplement: Supplementary file 3 — Additional file 3: Table S1. The normalized intensities and fold-changes of typical differentially expressed proteins and differentially abundant metabolites. [file 12870_2022_3547_MOESM3_ESM.docx]

Table S1 The normalized intensities and fold-changes of typical differentially expressed proteins and differentially abundant metabolites

| **Proteome** | | | | | | | | |
| --- | --- | --- | --- | --- | --- | --- | --- | --- |
| **Proteins** | **HD** | **5%RWC** | **RH-1h** | **RH-12h** | **5%RWC vs HD** | | **RH-12h vs 5%RWC** | |
|  | **Normalized intensities** | | | | **Fold-change** | ***P*-value** | **Fold-change** | ***P*-value** |
| **Antioxidant metabolism** | | | | | | | | |
| ascorbate peroxidase (APX) | 4.40E+07 | 8.69E+07 | 5.30E+07 | 5.69E+07 | 1.97 | 0.034213 | 0.6553 | 0.087549 |
| Monodehydroascorbate reductase 1 (MDHAR1) | 1.45E+08 | 2.18E+08 | 1.88E+08 | 1.64E+08 | 1.51 | 0.030909 | 0.7523 | 0.091398 |
| Monodehydroascorbate reductase 2 (MDHAR2) | 2.60E+07 | 4.23E+07 | 3.85E+07 | 3.19E+07 | 1.63 | 0.014842 | 0.7544 | 0.137558 |
| glutathione dehydrogenase/transferase (DHAR) | 1.20E+05 | 1.32E+06 | 1.00E+06 | 4.06E+05 | 11.00 | 0.000283 | 0.3066 | 0.006389 |
| glutathione reductase 1 (GSR1) | 4.83E+06 | 1.58E+07 | 9.50E+06 | 7.75E+06 | 3.27 | 0.000214 | 0.4914 | 0.003126 |
| glutathione reductase 2 (GSR2) | 6.91E+06 | 1.41E+07 | 8.11E+06 | 8.88E+06 | 2.05 | 0.012046 | 0.6282 | 0.036292 |
| glutathione S-transferase (GST) | 6.61E+05 | 2.03E+06 | 1.25E+06 | 1.89E+06 | 3.08 | 0.029431 | 0.9321 | 0.874488 |
| S-adenosylmethionine synthetase (metK) | 1.51E+08 | 2.46E+08 | 1.61E+08 | 1.45E+08 | 1.63 | 0.010023 | 0.5914 | 0.006592 |
| polyamine oxidase (MPAO) | 2.43E+06 | 5.24E+06 | 4.82E+06 | 3.58E+06 | 2.16 | 0.019610 | 0.6843 | 0.159296 |
| **Photosynthesis** | | | | | | | | |
| F-type H+-transporting ATPase subunit gamma (ATPF1G) | 2.78E+07 | 5.59E+06 | 2.72E+07 | 2.11E+07 | 0.2013 | 0.001820 | 3.78 | 0.036717 |
| F-type H+/Na+-transporting ATPase subunit beta (ATPF1B) | 6.43E+08 | 1.51E+05 | 1.51E+05 | 1.51E+05 | 2.35E-04 | 0.000055 | 1.00 | NA |
| apocytochrome f (petA) | 9.43E+07 | 5.28E+07 | 7.92E+07 | 6.65E+07 | 0.5596 | 0.004353 | 1.26 | 0.145782 |
| ferredoxin--NADP+ reductase (petH) | 2.37E+08 | 1.30E+08 | 9.72E+07 | 2.65E+08 | 0.5480 | 0.004670 | 2.04 | 0.020226 |
| photosystem I subunit VII (psaC) | 2.41E+07 | 9.91E+06 | 1.52E+07 | 1.60E+07 | 0.4116 | 0.015245 | 1.62 | 0.002267 |
| photosystem II oxygen-evolving enhancer protein 1 (psbO) | 1.70E+08 | 6.23E+07 | 1.53E+08 | 1.48E+08 | 0.3659 | 0.003860 | 2.37 | 0.001131 |
| photosystem II P680 reaction center D2 protein (psbD) | 6.99E+07 | 3.57E+07 | 5.66E+07 | 4.51E+07 | 0.5104 | 0.023484 | 1.26 | 0.316238 |
| **Carbon fixation in photosynthetic organisms** | | | | | | | | |
| glyceraldehyde 3-phosphate dehydrogenase (GAPDH) | 1.53E+08 | 4.56E+07 | 1.34E+08 | 1.60E+08 | 0.2988 | 0.007952 | 3.51 | 0.002375 |
| phosphoglycerate kinase (PGK) | 2.14E+08 | 1.38E+08 | 1.68E+08 | 1.73E+08 | 0.6448 | 0.024851 | 1.25 | 0.215055 |
| ribulose-bisphosphate carboxylase large chain (rbcL) | 4.05E+09 | 2.28E+09 | 2.61E+09 | 3.29E+09 | 0.5617 | 0.000231 | 1.44 | 0.070441 |

| **metabolome** | | | | | | | | | | |
| --- | --- | --- | --- | --- | --- | --- | --- | --- | --- | --- |
| **Metabolites** | **HD** | **5%RWC** | **RH-1h** | **RH-12h** | **5%RWC vs HD** | | **RH-12h vs 5%RWC** | | **RH-12h vs HD** | |
|  | **Normalized intensities** | | | | **Fold-change** | ***P*-value** | **Fold-change** | ***P*-value** | **Fold-change** | ***P*-value** |
| **Antioxidant metabolism** | | | | | | | | | | |
| Ascorbate (AsA) | 2.65 | 1150.88 | 8.98 | 13.08 | 433.59 | 0.000143 | 0.0114 | 0.000156 | 4.93 | 0.012681 |
| Dehydroascorbic acid (DHA) | 3.15E+08 | 5.07E+08 | 4.45E+08 | 4.91E+08 | 1.61 | 0.000023 | 0.9681 | 0.760859 | 1.56 | 0.002820 |
| Glutathione (GSH) | 1.81E+06 | 8.16E+07 | 3.99E+05 | 1.08E+06 | 45.13 | 0.012225 | 0.0132 | 0.011605 | 0.5961 | 0.000978 |
| gamma-Glutamylcysteine | 8.50E+08 | 5.63E+08 | 8.01E+08 | 7.52E+08 | 0.6632 | 0.000976 | 1.33 | 0.005178 | 0.9643 | 0.384176 |
| Glycine | 1.78E+03 | 9.59E+02 | 1.76E+03 | 1.77E+03 | 0.5382 | 7.78E-06 | 1.85 | 0.001275 | 0.9941 | 0.950489 |
| S-Adenosylmethionine (SAM) | 1.01E+07 | 6.15E+07 | 3.48E+07 | 1.03E+07 | 6.07 | 0.005852 | 0.1669 | 0.007799 | 1.01 | 0.977971 |
| Methionine | 2.68E+08 | 1.67E+08 | 3.53E+08 | 4.77E+08 | 0.6238 | 0.000080 | 2.86 | 0.000153 | 1.78 | 0.004541 |
| Spermidine (SPD) | 8.36E+07 | 4.74E+08 | 4.54E+07 | 5.19E+07 | 5.66 | 4.50E-08 | 0.1097 | 1.85E-08 | 0.6212 | 0.001985 |
| spermine (SPM) | 3.55E+06 | 2.59E+06 | 2.93E+06 | 6.59E+06 | 0.7281 | 0.287766 | 2.55 | 0.052021 | 1.85 | 0.112684 |
| putrescine (PUT) | 3.04E+07 | 1.93E+07 | 5.86E+06 | 5.99E+06 | 0.6332 | 0.326391 | 0.3107 | 0.084378 | 0.1967 | 0.014610 |
| **Carbon fixation in photosynthetic organisms** | | | | | | | | | | |
| Fructose 6-phosphate | 5.12E+07 | 1.56E+07 | 9.65E+07 | 1.79E+08 | 0.3047 | 0.000111 | 11.49 | 3.57E-10 | 3.50 | 1.19E-08 |
| D-Ribulose 5-phosphate | 2.32E+08 | 2.27E+07 | 5.85E+08 | 4.70E+08 | 0.0975 | 0.000063 | 20.76 | 0.003348 | 2.02 | 0.093456 |
| D-Erythrose 4-phosphate | 4.53E+07 | 1.92E+06 | 2.28E+07 | 2.29E+07 | 0.0424 | 1.93E-15 | 11.93 | 1.23E-06 | 0.5054 | 1.24E-06 |
| Fructose 1,6-bisphosphate | 1.52E+09 | 3.36E+08 | 3.03E+08 | 1.36E+08 | 0.2211 | 0.001073 | 0.4038 | 0.002818 | 0.089 | 0.000319 |
| Sedoheptulose 7-phosphate | 1.77E+08 | 5.27E+07 | 4.63E+08 | 3.48E+08 | 0.2978 | 0.000214 | 6.60 | 0.004120 | 1.97 | 0.076111 |
| **Osmotic regulaters** | | | | | | | | | | |
| Myo-Inositol | 158.77 | 308.16 | 195.02 | 153.48 | 1.94 | 1.44E-06 | 0.4981 | 9.09E-07 | 0.9667 | 0.446853 |
| Sorbitol | 42.66 | 126.46 | 7.55 | 49.45 | 2.96 | 0.015524 | 0.3911 | 0.021482 | 1.16 | 0.394371 |
| Threitol | 44.64 | 107.13 | 53.23 | 65.70 | 2.40 | 1.29E-06 | 0.6133 | 4.18E-06 | 1.47 | 0.002716 |
| Xylitol | 35.53 | 74.14 | 31.52 | 33.09 | 2.09 | 1.99E-06 | 0.4464 | 1.15E-06 | 0.9314 | 0.029698 |
| Sucrose | 1.85 | 1.69 | 18.22 | 1.90 | 0.9141 | 0.842502 | 1.12 | 0.696990 | 1.02 | 0.960236 |
